# Pattern Visual Evoked Potentials Elicited by Organic Electroluminescence Screen

**DOI:** 10.1155/2014/606951

**Published:** 2014-08-14

**Authors:** Celso Soiti Matsumoto, Kei Shinoda, Harue Matsumoto, Hideaki Funada, Kakeru Sasaki, Haruka Minoda, Takeshi Iwata, Atsushi Mizota

**Affiliations:** ^1^Department of Ophthalmology, Teikyo University School of Medicine, Kaga 2-11-1, Itabashi-ku, Tokyo 173-8605, Japan; ^2^Matsumoto Eye Clinic, 50-2 Takagaki, Awa-cho, Awa-shi, Tokushima 771-1705, Japan; ^3^Engineering Department, Tomey Corporation, 2-11-33 Noritakeshinmachi, Nishi-ku, Nagoya-shi, Aichi 451-0051, Japan; ^4^National Institute of Sensory Organs, National Tokyo Medical Center, 2-5-1 Higashigaoka, Meguro-ku, Tokyo 152-8902, Japan

## Abstract

*Purpose*. To determine whether organic electroluminescence (OLED) screens can be used as visual stimulators to elicit pattern-reversal visual evoked potentials (p-VEPs). *Method*. Checkerboard patterns were generated on a conventional cathode-ray tube (S710, Compaq Computer Co., USA) screen and on an OLED (17 inches, 320 × 230 mm, PVM-1741, Sony, Tokyo, Japan) screen. The time course of the luminance changes of each monitor was measured with a photodiode. The p-VEPs elicited by these two screens were recorded from 15 eyes of 9 healthy volunteers (22.0 ± 0.8 years). *Results*. The OLED screen had a constant time delay from the onset of the trigger signal to the start of the luminescence change. The delay during the reversal phase from black to white for the pattern was 1.0 msec on the cathode-ray tube (CRT) screen and 0.5 msec on the OLED screen. No significant differences in the amplitudes of P100 and the implicit times of N75 and P100 were observed in the p-VEPs elicited by the CRT and the OLED screens. *Conclusion*. The OLED screen can be used as a visual stimulator to elicit p-VEPs; however the time delay and the specific properties in the luminance change must be taken into account.

## 1. Introduction

Cathode-ray tube (CRT) monitors have been used as visual stimulators to elicit pattern-reversal visual evoked potentials (p-VEPs). However, CRT has become less available in the market. As it has been extensively replaced by liquid crystal displays (LCD) as a television monitor and computer monitor, one might imagine that LCD may be good replacement for CRT as a visual stimulator for p-VEPs. But LCDs have an inherent problem as visual stimulators because they take several milliseconds for the crystal molecules to change their alignment to permit the light to pass through the polarizing filter of the LCD [1, 2] (http://www.sharp.co.jp/products/lcd/tech/s2_1.html). This causes a transient change of the mean luminance of the entire LCD screen at the time of the reversal, and this change in the luminance can elicit electroretinograms (ERGs) and flash VEPs. We named this phenomenon the flash effect [3]. The p-VEPs elicited by LCD screens have longer implicit times than those elicited by CRT screens due to several factors such as the total temporal differences between the LCD's electronic input and radiometric output signals, caused by the response time and the input lag, and the flash effect [4–7]. We have shown that the flash effect can be reduced by using 2 ms response time LCD screens and reducing the contrast of the checkerboard luminance pattern on the LCD screens [3, 7]. Because the properties of the luminance changes vary for individual LCD screens, this may restrict the use of LCD screens as a general standard visual stimulator to elicit p-VEPs.

The recently developed organic electroluminescence (OLED) screen has a faster response time than standard LCD screens [8, 9] and, thus, it may be suitable for a visual stimulator to elicit p-VEPs. The purpose of this study was to compare the luminance profile of OLED screen to that of a CRT screen and to evaluate the usefulness of OLED screen as a visual stimulator to elicit p-VEPs.

## 2. Subjects and Methods

### 2.1. Subjects

Fifteen eyes of 9 healthy volunteers who did not have any ocular diseases except for refractive errors were studied. There were nine women whose mean age was 22.0 ± 0.8 years (±standard deviation) with a range of 21–23 years. The guidelines of the Declaration of Helsinki were followed and the procedures used were approved by the Institutional Review Board of Teikyo University. An informed consent was obtained from all of the subjects after an explanation of the purpose of the study, procedures to be used, and possible complications.

### 2.2. Methods

#### 2.2.1. Measurement on Luminance of Single Check

To determine the time delay of each monitor, the luminance change of a single check was measured with a photodiode (S1133, Hamamatsu Photonics Co., Ltd., Hamamatsu, Japan). The photodiode was attached to the upper left corner of one check. The signal was amplified at 1 × 10^7^ by a photosensor amplifier C9329 (Hamamatsu Photonics Co., Ltd., Hamamatsu, Japan) with a band frequency from DC to 1.6 kHz.

In addition, the luminance at the 4 outer corners and one point at the center of the entire checkerboard screen was measured with a luminance meter (CA-100S, Konica Minolta Inc., Osaka, Japan). We confirmed that the variations in the luminance from the center to the periphery were within 20% for each of the monitor which complies with the standards of the ISCEV guidelines (Table 1) [10].

Although the luminance of the OLED screen could be set to be blacker than the other screens, it was set to be equal to that of the CRT screens.

The luminance and contrast of both the CRT and the OLED screens were matched. The contrast between the black and white checks was calculated with the Michelson contrast formula [11].

#### 2.2.2. Pattern-Reversal Stimuli

The visual stimulus was a black and white checkerboard generated either on a CRT screen (17 inches, 320 × 230 mm, S710, Compaq Computer Co., USA) or on an OLED screen (17 inches, 365.7 × 205.7 mm, PVM-1741, pixel dimensions, 1920 × 1080, Sony, Tokyo, Japan). Because the aspect ratio of the OLED screen did not match the checkerboard stimulus pattern, the checkerboard pattern of 800 × 600 pixels was created at the center of the OLED screen by an analogue-digital converter (CP-293 Cypress Technology Enterprises, Inc., CA, USA). An analogue-digital converter was used to connect the pattern generator (LE-4000, Tomey Corporation, Nagoya, Japan), that supports only analogue VGA interface, while the OLED display (PVM-1741, Sony, Tokyo, Japan) only supports HDMI (digital) interface. The OLED screen used is commercially available. The response time of the LCD screen was 2 ms for the LCD. Other investigators consider the response time to be the time required to change from gray to gray [2, 3].

The maximum contrast was 97% and the check size was 0.25 degrees at an observation distance of 70 cm. The reversal rate was 3.0 rev/sec. The resolution of each monitor was 800 × 600 pixels and the vertical frequency was 59.8 Hz.

#### 2.2.3. P-VEP Recordings

All recordings were performed under dim room lights of 104 lux and the subjects were preadapted to the room lighting before beginning the recordings. A small black fixation point was placed at the corner of the four central checks of the stimulus pattern and the subjects were instructed to fixate the point or, if the point was not visible, the center of the screen and to try not to blink. The subjects wore their best refractive correction and all recordings were monocular.

The recording electrode was placed on the inion (Oz) and the reference electrode was placed at Fz. The ground electrode was placed on the right earlobe. Signals were amplified 4,000 times (LE-4000, Tomey Corporation, Nagoya, Japan) and bandpass filtered from 1.0 to 100 Hz. The sampling rate was 1.0 kHz and one hundred twenty-eight responses were averaged. The recordings were performed at least twice to determine the repeatability. In addition, the measurements for each subject were performed twice within one week to determine the intermeasurement variability.

### 2.3. Data Analyses

The P2 amplitude was measured between the trough of N-75 to the peak of P-100 and the implicit times of N-75 (N1 implicit time) and P-100 (P2 implicit time) between the onset of the trigger and the trough of N-75 or peak of P-100. Student's* t*-tests were used to determine the significance of differences of each parameter. A *P* < 0.05 was taken to be significant.

## 3. Results

### 3.1. Luminance Changes of Checkerboard for Each Monitor

**Figure 1:**
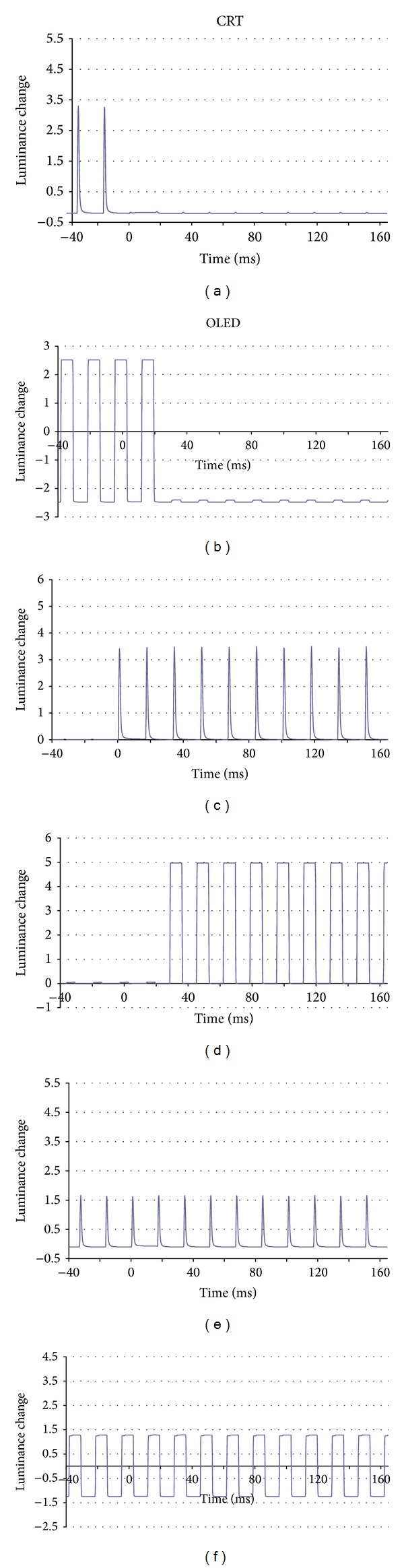
Changes in the average luminance of a single check of the cathode-ray tube (CRT) screen and the organic electroluminescence (OLED) screen during pattern reversal. There is no luminance change in the overall luminance across the screen because half of the checks are changing in the opposite direction. ((a), (c), and (e)) cathode-ray tube (CRT) screen shows burst of pulses and ((b), (d), and (f)) organic electroluminescence (OLED) screen shows rectangular-shaped luminance change. (a) Luminance changes of a single check from white to black of CRT screen. (c) Luminance changes of a single check from black to white of CRT screen. (e) Averaged luminance changes of the CRT screen. There is no change in the total luminance (*y*-axis) during time (*x*-axis). (b) Luminance changes of a single check from white to black of OLED screen. (d) Luminance changes of a single check from black to white of OLED screen. (f) Averaged luminance changes of OLED screen. There is no change in the total luminance (*y*-axis) during time (*x*-axis).

**Figure 2:**
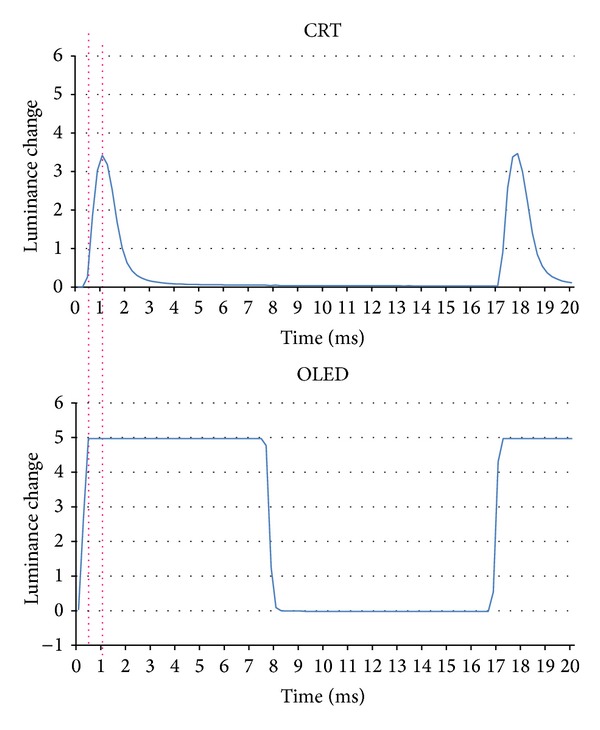
Luminance change of a single check during reversal of black to white. Short and constant delay as a response time was detected during the check reversal of approximately 1.0 ms in the CRT screen and approximately 0.5 ms in the OLED screen.

The changes in the luminance are plotted against time in Figure 1. A burst of pulses at 60 Hz was delivered to the CRT monitor and a square wave pulse was delivered to the OLED screen to change the luminance of the checks. The input lag, the time between the signal input to the screen to the time a change in luminance is detected, was 0.8 ms for the CRT and 28.4 ms for the OLED. The short and constant delay of the response time was detected during the check reversal to be approximately 1.0 ms for the CRT screen and approximately 0.5 ms for the OLED screen (Figure 2).

The luminance changes of the LCD screen (XL2410T, 23.6 inches, 570 × 347.4 mm BENQ Co., Taipei, Taiwan.) are shown in Supplemental Figure 1 (see Supplementary Material available online at http://dx.doi.org/10.1155/2014/606951). Nagy et al. reported that the p-VEPs elicited by LCD screens had longer implicit times than those elicited by CRT [6]. The delay was attributed to the total temporal differences between the LCD's electronic input and radiometric output signals, caused by the response time and the input lag. When referred to the trigger, the input lag was measured to be approximately 0.8 ms for the CRT and 28.6 ms for the OLED screens used in this study. The input lag is the time between the input signal leaving the video card and the image appearing on the screen [6, 12]. The reason for this lag is that the input signal is further processed at the display level before it appears on the screen. The image processing technologies and processing times can vary with the manufacturer, display type, and setup parameters, such as the resolution, color settings, and internal processes. Because the input lag was constant for the monitors used, it was subtracted from the implicit time in the analyses of the p-VEPs (see Section 3.2.).

### 3.2. Comparison of P-VEP Components between CRT and OLED Screens

**Figure 3:**
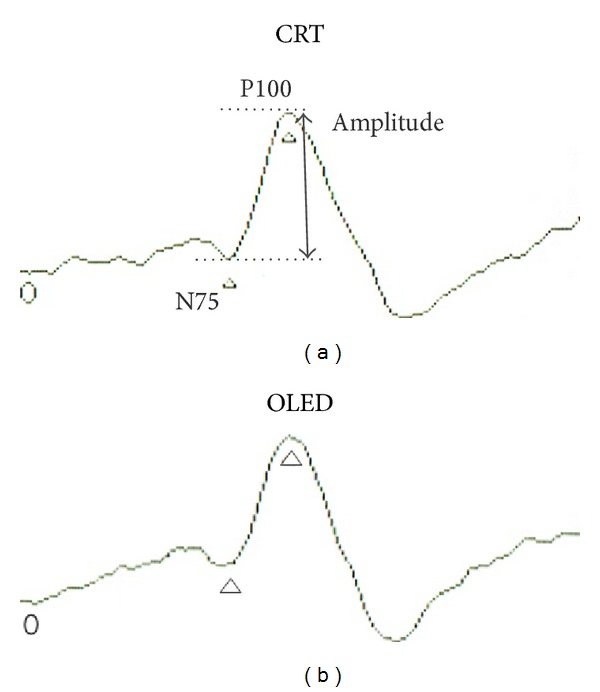
Representative waveform of p-VEP. P-VEP waveforms elicited by CRT (a) and OLED (b) screens.

**Figure 4:**
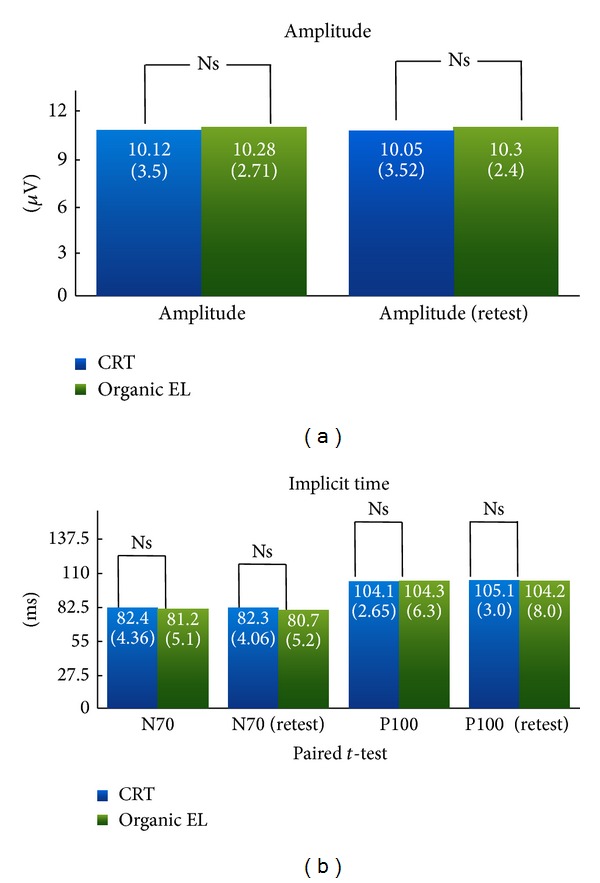
Comparisons of each parameter between the pattern VEPs (p-VEPs) elicited by CRT and by OLED screens. (a) No significant difference was found between the p-VEP P100 amplitude elicited by the OLED screen and that elicited by the CRT screen. (b) No significant difference was found in the implicit time of N75 elicited by the OLED screen to between the p-VEP elicited by the CRT and OLED screens as a stimulator. No significant difference was observed in the implicit times of N75 between the p-VEPs elicited by the CRT and the OLED screens as a stimulator. ns: not significant.

Reproducible VEPs were elicited from the patterns generated on each monitor (Figure 3, Table 2). The P100 amplitude and the N75 and P100 implicit times are plotted in Figures 4(a) and 4(b), respectively. When measuring the implicit time, the input lag of 28.4 msec for the OLED screen was subtracted from the measured times (see Section 3.1.). The differences in the P100 amplitudes between the responses elicited by each screen were not significant. When compared to the VEPs elicited by the CRT screen, the N75 and P100 implicit time by the OLED screen were not delayed.

## 4. Discussion

The ISCEV standard for p-VEPs (2009 update) [11] specifies that the luminance reversal of the black and white checks changes abruptly at a specific number of reversals/sec. It also states that there must be no overall change in the luminance of the screen which indicates that there are an equal number of light and dark elements in the display, and no transient luminance changes occur during the pattern reversal. At present, only CRT screens can meet these standards because LCD screens have an inherent time delay when the luminance reverses. Our earlier experiments showed that the time delay causes a transient luminance change which can evoke an ERG and a flash VEP [3]. The flash effect can be minimized by decreasing the contrast of the checks, but the contrast must be reduced to 65% to completely eliminate the luminance artifact when using the 5 ms response LCD screens (17 inches, 340 × 270 mm, RDT233WX, Mitsubishi, Tokyo, Japan) [3]. This lower contrast does not meet the ISCEV standard [11].

Another solution to minimize the flash effect is to use a LCD screen with a shorter response time. But ERGs could still be elicited when the 2 ms response LCD screen was covered with a diffuser. Thus, we reduced the contrast of the checkerboard pattern to decrease the flash effect as we did for 5 ms LCD screen [3, 7]. Our results showed that the flash effect was greatly reduced and ERGs were not elicited with 81% contrast. From these results, we concluded that a flash VEP can be eliminated by using a 2 ms response LCD screen with 81% contrast and that the 2 ms response LCD screen is a better substitute for the CRT screen as a stimulator for eliciting p-VEPs especially when the contrast was set at 81% [7]. But setting these conditions for the LCD screen is not easy especially in a clinic.

OLED displays have recently been used for digital displays in devices such as mobile phones, handheld games consoles, and personal digital assistances. Due to current difficulties in producing large size OLED screens and their relatively high cost, there are limited number of OLED television screens and computer monitors. But it is expected that they will become more easily available. Their characteristics have been evaluated [9, 13] and our results showed the feasibility of their use as visual stimulators to elicit p-VEPs.

The luminance changes measured with a photosensor were comparable between the OLED and CRT screens with very rapid rise and fall times of the black and white checks (Figure 1). But the pulses causing the luminance changes were basically different, rectangular for the OLED and flickering bursts in CRT.

Recently, the characteristics of an OLED screen (Sony PVM-2541, 24.5 in.; Sony Corporation, Tokyo, Japan) have been precisely measured from the viewpoint of its applicability to visual psychophysics [13, 14]. They stated that the tested OLED display had excellent luminance and color uniformity, excellent low luminance gradations, stable white and three primaries throughout the wide luminance range, wide color space (especially for saturated green), and rapid luminance rise/fall times. They state that when large enough OLED displays become available, they would be ideal for vision research because they provide self-illumination, rapid rise/fall luminance level performance, and high contrast images. However they also stated that the concept of one frame in the PVM-2541 is different from those in the LCD and CRT display, and it is unclear whether these differences will affect the perception of briefly presented stimuli.

Our results showed that the p-VEPs elicited by OLED screens were not significantly different from those elicited by conventional CRT screens. The amplitude of P-100 and implicit times of N-75 and P-100 were almost identical between two waves when the constant input lag was subtracted from the measurements of the p-VEP elicited by the OLED screen.

This study has several limitations. The property of the luminance change was different and its influence on the retinal and optic nerve responses was unknown. Investigating the influence of the different properties on the human visual system will be interesting but here we have just investigated the possibility of substituting CRT monitor with OLED monitor as a visual stimulator for p-VEP. We investigated a single LCD and a single OLED monitor but the input lag and response time are unique in LCD and OLED screens. Therefore, a better LCD screen or a better OLED monitor as a visual stimulator may be found with further investigations.

In conclusion, the OLED screen can be a better substitute for the CRT screen and also LCD screens as a stimulator for eliciting p-VEPs. However, it will be important to collect normative data because how the different luminance changes will affect human perception of briefly presented stimuli is unknown.

## Supplementary Material

Luminance change of a single check of a conventional 60 Hz liquid crystal (LCD) screen.

## Figures and Tables

**Table 1:** Mean luminance of pattern VEP white and black squares of the checkerboard in each screen.

Screen	Stimulus white (cd/m^2^) min. and max. (mean) luminance	Stimulus black (cd/m^2^) min. and max. (mean) luminance
CRT	149, 158 (153)	3, 3 (3)
Organic LED	149, 154 (151)	3, 3 (3)

**Table 2:** Comparison of p-VEP parameters between two groups.

	Amplitude (uV)		Implicit time (ms)			
			N75		P100	
	Test	Retest	Test	Retest	Test	Retest
CRT	10.12 ± 3.50	10.05 ± 3.52	82.4 ± 4.36	82.3 ± 4.06	104.1 ± 2.65	105.1 ± 3.0
OLED	10.28 ± 2.71	10.30 ± 2.40	81.2 ± 5.10	80.7 ± 5.20	104.3 ± 6.3	104.2 ± 8.0

*P* value (*t*-test)	0.9937	0.9883	0.1741	0.0661	0.1718	0.3735
CI (difference of two groups)	−5.17~5.20	−4.12~4.16	−1.70~7.17	−0.2~4.33	−1.47~6.27	−3.1~6.9

P-VEP: pattern visual evoked potentials, CRT: cathode-ray tube screen, OLED: organic electroluminescence screen, and CI: confidential interval.
