# Supplementary material for: Pattern Visual Evoked Potentials Elicited by Organic Electroluminescence Screen
Source: Biomed Res Int. 2014 Aug 14;2014:606951. doi: 10.1155/2014/606951 (PMC4147363; doi:10.1155/2014/606951)
Supplement: Supplementary file 1 — Luminance change of a single check of a conventional 60 Hz liquid crystal (LCD) screen. [file 606951.f1.zip › mat.606951.v2.pdf]

Supplemental Figure

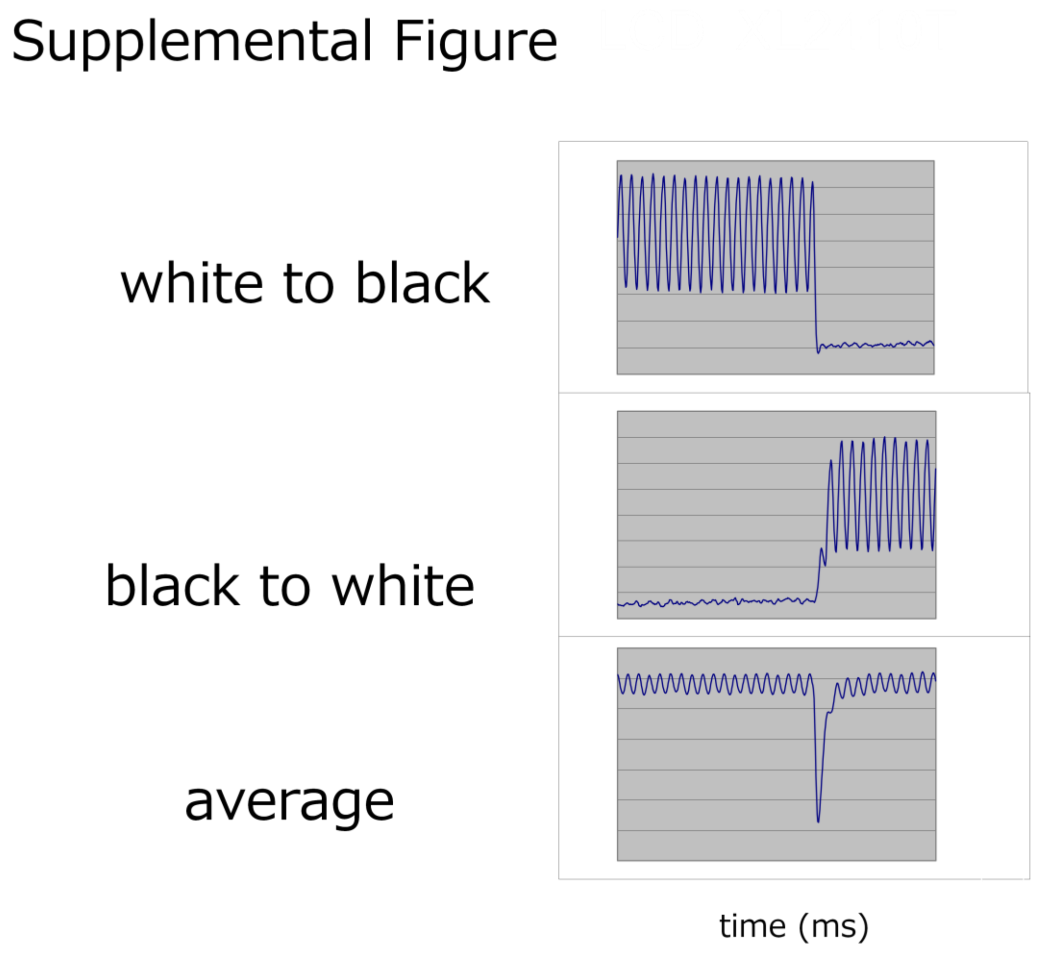

Supplemental Figure 1. Luminance change of a single check of a conventional 60 Hz liquid crystal (LCD) screen. There is an abrupt change of the luminance (y axis) at the time of reversal of the checkerboard (x axis). The response time of the LCD (XL2410T, 23.6 inch, 570x347.4 mm BENQ Co, Taipei, Taiwan) screen was 2 ms for the LCD according to the specifications of the manufacturer. The response time is defined as the time it takes one pixel to turn from black to white.
