# Supplementary figures and images for: Pattern Visual Evoked Potentials Elicited by Organic Electroluminescence Screen
Source: Biomed Res Int. 2014 Aug 14;2014:606951. doi: 10.1155/2014/606951 (PMC4147363; doi:10.1155/2014/606951)

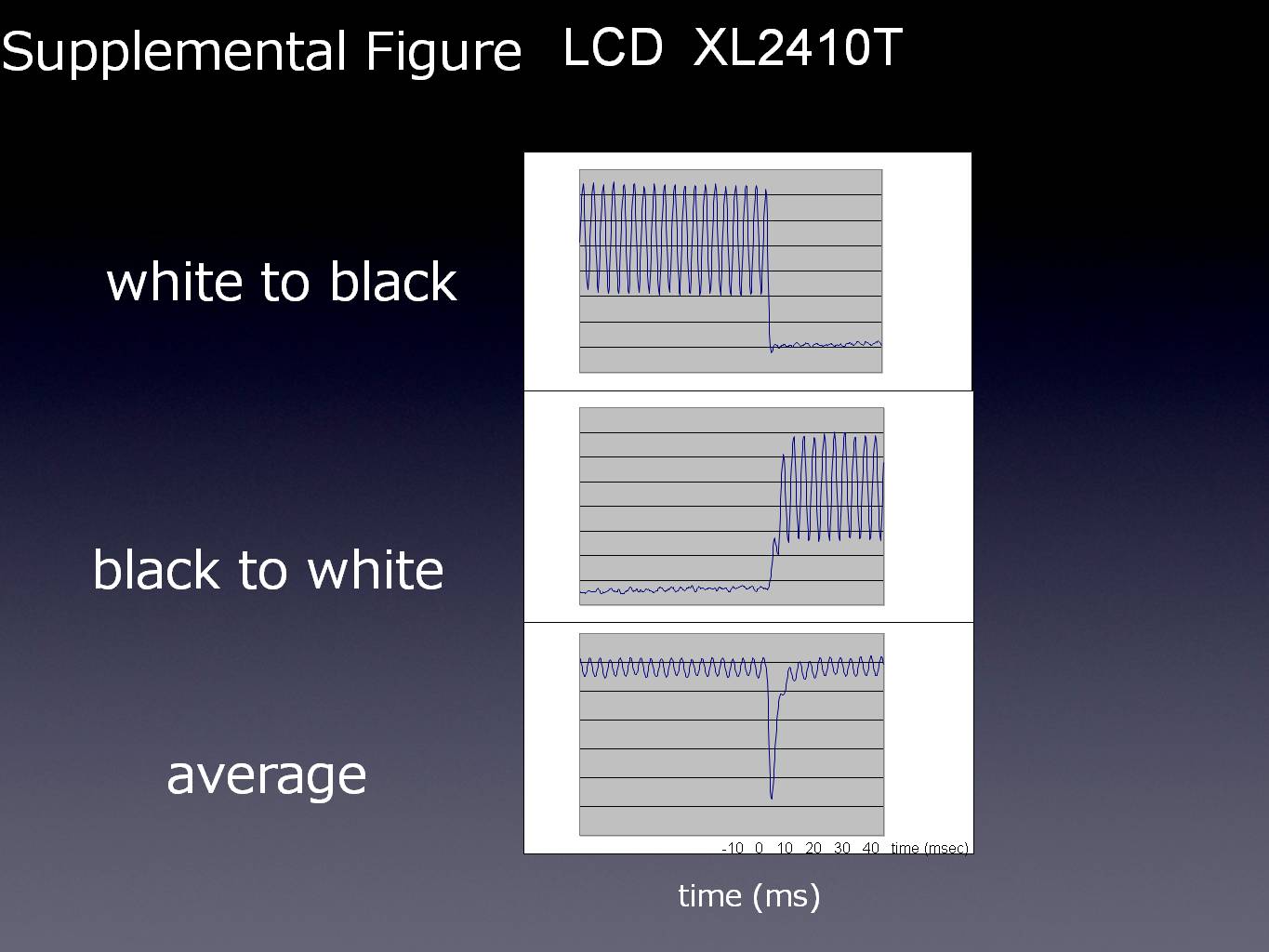

Supplement: Supplementary file 1 — Luminance change of a single check of a conventional 60 Hz liquid crystal (LCD) screen. [file 606951.f1.zip › mat.606951.v1.jpg]
